# Mothers’ Impressions and Beliefs About Taking a Booster Dose for COVID-19 Vaccine During Pregnancy and Lactation

**DOI:** 10.7759/cureus.32561

**Published:** 2022-12-15

**Authors:** Esra' O Taybeh, Rawan Alsharedeh, Shereen Hamadneh

**Affiliations:** 1 Department of Applied Pharmaceutical Sciences, School of Pharmacy, Isra University, Amman, JOR; 2 Department of Pharmaceutical Sciences, School of Pharmacy, Yarmouk University, Irbid, JOR; 3 Department of Maternal and Child Health, School of Nursing, Al al-Bayt University, Mafraq, JOR

**Keywords:** booster vaccine, pregnancy, maternal and child health, lactation, covid-19 vaccine

## Abstract

Objectives: This study aimed to explore perceptions and willingness to get coronavirus disease 2019 (COVID-19) booster vaccination among pregnant and lactating women in Jordan.

Methods: A cross-sectional study using a 29-item web-based questionnaire was conducted. Sociodemographic characteristics, vaccine acceptance, confidence in the booster dose of COVID-19 vaccine, perception of risk for COVID-19, and acceptance to participate in COVID-19 booster vaccine clinical trials were prospectively evaluated. Logistic regression was used to identify factors that might affect the participants’ acceptance of a COVID-19 vaccine and their willingness to enroll in clinical trials of a booster dose of COVID-19 vaccine.

Results: Among all participants (pregnant and lactating women, n = 584), 328 (56.2%) intended to receive the booster dose of the COVID-19 vaccine. Predictors of booster dose acceptance were a medical-related degree (OR 1.62, CI 1.06-2.5, p = 0.028), income (OR 0.677, CI 0.52-0.88, p = 0.004), living residency (OR 0.44, CI 0.32-0.60, p < 0.001), knowing pregnant/lactating women previously infected with infectious microbe (OR 1.539, CI 1.07-2.23, p = 0.022), commitment to immunization for children (OR 3.01, CI 1.03-8.82, p = 0.044), receiving an influenza vaccine (OR 1.46, CI 1.04-2.05, p = 0.031), and worried about infectious microbes (OR 1.32, CI 1.15-1.52, p < 0.001). Among participants, only 22.9% were willing to participate in clinical trials of the booster dose of COVID-19 vaccine. The biggest motivator for participation was the participants’ desire to help find the best vaccine during pregnancy/lactation (57.5%) while the main barrier towards participation was not wanting to expose themselves and their babies to more side effects (88.0%).

Conclusion: This study reported reasonable acceptance of vaccination in a sample of pregnant/lactating women. Vaccination hesitancy for the booster dose was in-line with similar studies on the primary series around the globe, but the willingness to participate in clinical trials was lower than non-pregnant/non-lactating women.

## Introduction

Pregnant women with coronavirus disease 2019 (COVID-19) infection are at increased risk for pregnancy complications, such as preterm birth and pregnancy loss, and neonatal intensive care unit admission [1]. Moreover, pregnant women may be at higher risk for COVID-19-related scenarios, such as intensive care unit admissions, mechanical ventilation, extracorporeal membrane oxygenation, and death compared with non-pregnant women [2].

Due to limited data concerning maternal-infant transmission of COVID-19 during breastfeeding, literature on breastfeeding management in confirmed COVID-19 mothers did not recommend breastfeeding in suspected and uncured cases and also while taking lopinavir/ritonavir treatment [3]. In addition, the Union of European Neonatal and Perinatal Societies advises mothers who are too sick to manage their babies separately with freshly expressed breastmilk to avoid any harm to the babies [4].

Vaccination during pregnancy and lactation is common to prevent both maternal and infant morbidity caused by other infectious diseases, such as influenza and pertussis. The clinical data on safety and efficacy of such vaccines are abundant [5,6]. However, pregnant and lactating women were excluded from the initial COVID-19 vaccine trials worldwide; thus, data to guide vaccine administration to such groups are lacking. The Centers for Disease Control and Prevention (CDC), the Society for Maternal-Fetal Medicine, the American College of Obstetricians and Gynecologists (ACOG), and the American Academy of Pediatrics have each issued guidance supportive of offering COVID-19 vaccines to pregnant women [7-9]. The Academy of Breastfeeding Medicine states that potential risks of COVID-19 infection should be weighed against the potential benefits of neonatal protection via passive transfer of antibodies from breast milk [10].

Additionally, in 2021, the ACOG recommended booster doses (third vaccination dose) for pregnant and post-partum women due to their increased risk of COVID-19-related consequences. Previous trials on pregnant women found that booster dose was associated with higher maternal antibody levels and thus lower risk for severe COVID-19-related outcomes [11] as well as a stronger level of protection for young infants compared to the second dose [12].

Even though recent guidance indicates that COVID-19 vaccines including booster doses should not be withheld from pregnant and lactating women, there is no consensus regarding vaccination during pregnancy/lactation and vaccine hesitancy among women still existed.

In January 2021, Jordan began its vaccination campaign against COVID-19, with healthcare workers and the elderly prioritized for the first shots. Thereafter, in September 2021, the Jordan Ministry of Health authorized the booster shot for people 60 years old and above, the immunocompromised, including those with chronic illnesses, and health workers [13]. The present work was performed at the time of launching the booster dose to public and aimed to explore perceptions and willingness to get COVID-19 booster vaccination among pregnant and lactating women in Jordan. These data might be used to address vaccine hesitancy and refusal driven by knowledge gaps since, until recently, there was a misunderstanding and insufficient knowledge about COVID-19 risks, management, and vaccination during pregnancy and lactation among women in Jordan [14].

## Materials and methods

This was a cross-sectional study conducted from November 2021 to January 2022 among pregnant and lactating women in Jordan. The sample population was a convenience sample targeted through social media platforms, i.e., Facebook groups. A minimum of 383 patients based on maximum annual deliveries of 80,000, 5% margin of error, 95% confidence level, and 50% response distribution were required. Any pregnant and lactating woman living in Jordan at the time of the study was eligible to participate. A detailed explanation about the study was provided to potential participants and consent about anonymity of the survey and voluntary participation was then obtained for each participant. The used platform allowed participants to move through sections only when their answers were completely obtained. The study was approved by the Ethical Approval Committee of the Institutional Review Board of Yarmouk University (approval number: IRB/2021/2).

The questionnaire of this study was structured based on a broad literature search using MEDLINE/PubMed and Google Scholar. Most relevant articles have been retrieved and then the questionnaire was designed [15-18]. The questionnaire was evaluated by a group of experts, then it was piloted on 30 subjects to provide feedback about the relevance, clarity, and comprehensibility of the questions (Appendix).

The survey consisted of 29 questions. Questions 1-15 captured demographics and current health status. Questions 16-19 were about the acceptance of vaccines in general. Questions 20-22 were about confidence in the COVID-19 booster vaccine. Questions 23-26 queried the perceived risks of the virus. Question 27 queried whether the participant was willing to participate in a COVID-19 vaccine (booster dose) clinical trial during pregnancy/lactation. Questions 28 and 29 were detailed questions that evaluated motivations that may encourage and barriers that may discourage participation in a clinical trial.

The survey data was analyzed using social science software, SPSS® version 26 (Chicago, IL: SPSS® Inc.). The mean, standard deviation, frequencies, and percentages were used for continuous and categorical variables as needed. Analytical graphs were used as appropriate. Logistic regression was used to identify factors that might affect the participants’ acceptance of a COVID-19 vaccine and their willingness to enroll in clinical trials of a booster dose of COVID-19 vaccine. Statistical significance was at a p < 0.05.

## Results

Demographic characteristics

Among 584 respondents, 37.3% (n = 218) and 59.9% (n = 350) were pregnant and lactating, respectively, at the time of taking the survey (Table 1). The mean age of all respondents, both pregnant and lactating mothers, was 29.7 years (SD = 4.8). Among participating women, 87% (n = 508) had at least one child. The majority had a Bachelor’s degree (68.2%) and 23.8% (n = 139) had a health program degree.

**Table 1:** Sociodemographic characteristics of participants (n = 584). *Other means separated, divorced, widowed. COVID-19: coronavirus disease 2019

Character		Frequency (%)
Age (mean, range)		29.7, 17-47
Educational level	Less than diploma	50 (8.6%)
	Diploma	47 (8.0%)
	Bachelor's	398 (68.2%)
	Postgraduate	89 (15.2%)
Having a health-related degree	Yes	139 (23.8%)
	No	445 (76.2%)
Monthly income	Less than or equal to 500 JD	356 (61.0%)
	501-1,000 JD	171 (29.3%)
	1,001-2,000 JD	45 (7.7%)
	More than 2,000 JD	12 (2.1%)
Marital status	Married	574 (98.3%)
	Other*	10 (1.7%)
Nationality	Jordanian	554 (94.9%)
	Non-Jordanian	30 (5.1%)
Place of residency	North Jordan	269 (46.1%)
	Middle Jordan	286 (49.0%)
	South Jordan	29 (5.0%)
Having chronic diseases	Yes	14 (2.4%)
	No	570 (97.6%)
Pregnancy/lactation status	Pregnant mother	218 (37.3%)
	Lactating mother	350 (59.9%)
	Both	16 (2.7%)
Total children number	0	76 (13.0%)
	1	183 (31.3%)
	2	145 (24.8%)
	3	103 (17.6%)
	4 or more	77 (13.2%)
Were you infected with COVID-19 before pregnancy?	Yes	61 (10.4%)
	No	523 (89.6%)
Were you infected with COVID-19 during pregnancy/lactation?	Yes	132 (22.6%)
	No	452 (77.4%)
Has someone pregnant/lactating you know been infected with COVID-19?	Yes	381 (65.2%)
	No	203 (34.8%)
Took the vaccine before pregnancy	First dose	21 (3.6%)
	Both doses	21 (3.6%)
	No	542 (92.8%)
Took the vaccine during pregnancy/lactating	First dose	70 (12.0%)
	Both doses	32 (5.5%)
	No	482 (82.5%)
Had side effects	No side effects	12 (11.8%)
	Mild to moderate side effects	90 (15.4%)
	Severe side effects	0 (0.0%)

General vaccines acceptance

The majority of participants (n = 565) stated their likelihood to vaccinate their children with routine national immunization program. In addition, the results showed that 84.1% and 84.6% believed that such vaccines are safe and effective, respectively. On the other hand, only 26.4% (n = 154) of participants received a previous seasonal influenza vaccine.

Confidence in COVID-19 vaccine booster dose

**Figure 1:**
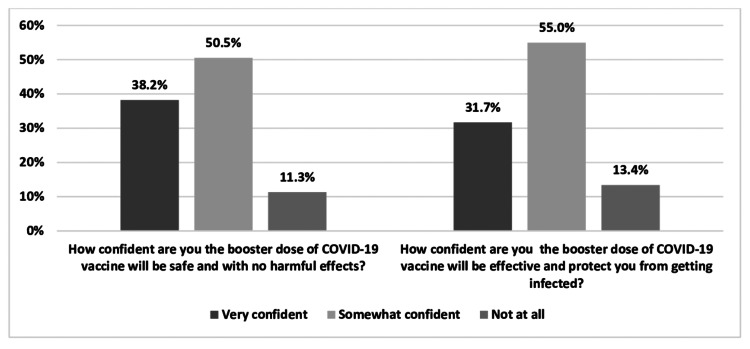
Booster dose of COVID-19 vaccine confidence level among pregnant/lactating women. COVID-19: coronavirus disease 2019

Among participants, 56.2% (n = 328) intended to receive the third dose of COVID-19 vaccination during their pregnancy/lactation period if efficacy and safety were achieved. Figure 1 shows the confidence levels of participants in a COVID-19 booster dose vaccine.

Perceived risk/precautions of the virus

Women’s level of worry about COVID-19 during pregnancy/lactation in the present study was high; 64.0% (n = 374) thought that they perceived a risk to catch COVID-19 during pregnancy/lactation. In regard to commitment to safety measures, 55.0% (n = 321) totally complied with face masks, social distancing guidelines, and other prevention measures. Despite this, only 15.8% seriously followed COVID-19 news (TV, newspaper, news websites, and social media).

Acceptance to participate in a third dose COVID-19 vaccine clinical trial

**Figure 2:**
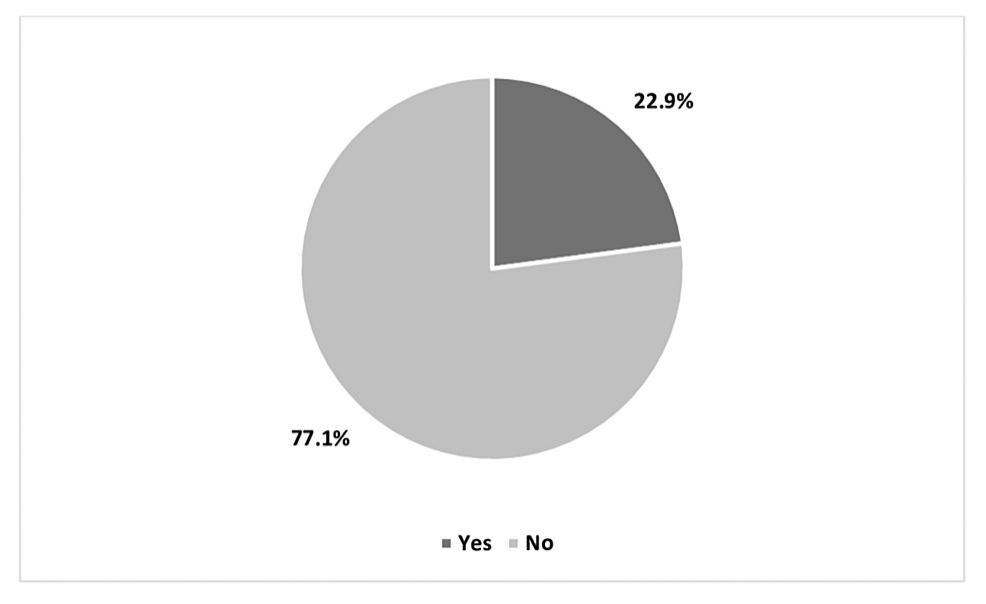
Willingness to participate in the third dose COVID-19 vaccine clinical trial during pregnancy/lactation. COVID-19: coronavirus disease 2019

Regarding participants’ acceptance to enroll in a COVID-19 vaccine clinical trial during pregnancy/lactation, the answers demonstrated that only 22.9% of participants (n = 134) were willing to participate in clinical trials while the remaining 77.1% (n = 450) refused (Figure 2).

**Figure 3:**
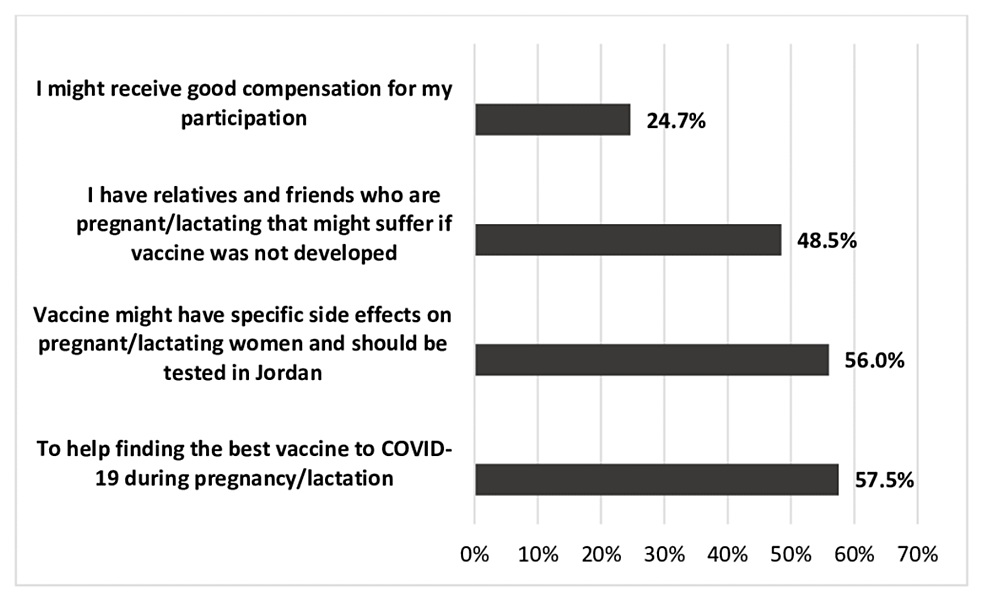
Motivators for the participation in booster dose of COVID-19 vaccine clinical trials during pregnancy/lactation. COVID-19: coronavirus disease 2019

The motivators that encouraged the participants to enroll in a booster dose vaccine clinical trial are presented in Figure 3. Participants' desire to help find the best vaccine for COVID-19 during pregnancy/lactation was the main motivator to participate (n = 336). On the other hand, only 24.7% (n = 144) would participate to receive compensation.

**Figure 4:**
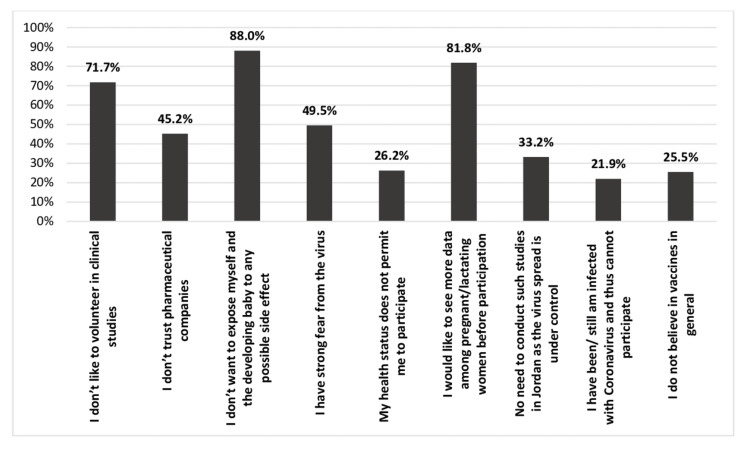
Barriers against the participation in booster dose of COVID-19 vaccine clinical trials during pregnancy/lactation. COVID-19: coronavirus disease 2019

Barriers to enroll in the booster doses of COVID-19 vaccine clinical trial are illustrated in Figure 4. Most of the participants did not want to expose themselves and their developing babies to any possible adverse effects (n = 514), and around 478 revealed that they would like to see more safety and effectiveness data about the vaccine among pregnant/lactating women and their babies before participation. Also, 419 of them reported that they would not like to volunteer in clinical studies either during pregnancy or lactation.

Predictors of booster dose of COVID-19 vaccine acceptance

The associations between the booster dose of COVID-19 vaccine acceptance during pregnancy/lactation and potential predictors showed that the factor of monitoring the COVID-19 news on media was slightly linked to booster dose acceptance. On the other hand, the strongest predictors of COVID-19 vaccine booster dose acceptance were among women who are having a health-related degree, have higher income, living in middle or South Jordan, being a lactating woman, know pregnant/lactating women previously infected with COVID-19, committed to routine immunization for children, receiving seasonal influenza vaccines, and worried about COVID-19 (Table 2).

**Table 2:** Logistic regression analysis of predictors of COVID-19 vaccine booster dose acceptance. *Did not miss any vaccine of the national vaccination schedule. **P-value less than 0.05 was considered significant. OR: odds ratio; CI: confidence interval; COVID-19: coronavirus disease 2019

Variable	OR	95% CI	p-Value**
Medical related degree	1.622	1.06-2.50	0.028
Income	0.677	0.52-0.88	0.004
Area of residency	0.435	0.32-0.60	0.000
Knowing infected pregnant/lactating woman	1.539	1.1.07-2.23	0.022
Commitment to child vaccine*	3.011	1.03-8.82	0.044
Receiving flu vaccines	1.457	1.04-2.05	0.031
Worried about COVID-19	1.324	1.15-1.52	0.000

## Discussion

Geographic variations in the acceptance rate of COVID-19 vaccination among pregnant and lactating women were reported in the literature. The acceptance level of COVID-19 vaccine in Mexico and India was above 80% for pregnant women. Whereas in the United States, Russia, and Australia, pregnant women expressed lower acceptance (below 45%) [15]. However, data on the endorsement of COVID-19 booster doses among pregnant and lactating women are scarce and it was not studied in the Middle East region. The acceptance rate of the booster dose of COVID-19 vaccine of 56.2% in the present study was consistent with the acceptance of influenza, tetanus, diphtheria, and pertussis vaccines recommended in pregnancy [19,20].

The strongest predictors of COVID-19 vaccine booster dose acceptance among participants were among women who are having a health-related educational level, higher income, living in Middle or South Jordan, being a lactating rather than pregnant woman, knowing pregnant/lactating women previously infected with COVID-19, committed to routine immunization for children, received seasonal influenza vaccines, and worried about COVID-19.

Although evidence confirming elevated risk from COVID-19 infection in pregnancy was reported, pregnant women have been excluded from most studies of COVID-19 treatments as well as from COVID-19 vaccine trials [21]. This exclusion could be the encouragement needed for pregnant/lactating women with a health-related profession to accept COVID-19 vaccines. It has been previously reported that some professions, such as dentists, nurse midwives, mental health counselors, and therapists had higher associations of vaccine acceptance [16].

A wide range of vaccine acceptance rate was previously reported in many countries based on income; those who live in low-income countries were found to have lower acceptance rates than those who live in higher-income countries [17]. These results on the primary series of COVID-19 vaccine are consistent with the findings of our study on the booster doses where women living within high-income families were more likely to accept.

The present study found that mothers who live in the middle and south regions of Jordan had higher acceptance rates of a booster dose of COVID-19 vaccine. This could be justified by their belief in the importance of vaccines to people living in these tourist sites of the country as tourism destinations were negatively affected by the COVID-19 pandemic, with almost 100 travel agencies, 80% of hotels (three stars and below), and 800 restaurants and local cafes closed. Moreover, around 14,000 employees lost their sources of income, many of them live and work at tourist sites [22].

Our results showed that pregnant respondents were more likely to decline vaccination compared to lactating respondents. This finding is in line with a previously published survey on vaccine acceptance [16]. The later study found that non-pregnant participants were most likely to accept vaccines with breastfeeding participants the second most likely while pregnant participants had the lowest rate of vaccine acceptance. This could be the result of pregnant women worrying that the vaccine could cause harm to them or their fetuses. In addition, those who know pregnant/lactating women previously infected with COVID-19 had higher vaccine booster acceptance. This could be due to their concerns about having the same experience and the possibility that they will be at risk of contracting COVID-19 or becoming severely ill from it.

Globally, routine immunization for children is relatively high and for instance, 83% of the world’s children receive full doses of the diphtheria-tetanus-pertussis vaccine (DTP3) every year [23]. Similar results were found in the present study where 96.7% indeed have their children fully vaccinated; without missing any obligatory ones. This indicates that women who accept their children to be vaccinated value vaccines including booster doses. Also, those who receive seasonal flu vaccines had a higher acceptance of a COVID-19 vaccine booster.

The results also showed that the perceived risk of COVID-19 for pregnant/lactating women, as measured by their worries toward COVID-19 was a strong predictor for acceptance of having COVID-19 vaccine booster dose. Even though this factor is being influenced by the current pandemic status, it seems that it has previously affected vaccine hesitancy [24].

The study also showed that a low proportion (23%) of participants had willingness to participate in clinical trials for COVID-19 vaccine; which was lower than the rate reported in France and China [18,25]. The top reason for pregnant/lactating women to refuse enrollment in clinical trials for COVID-19 booster vaccination was their worries of being further exposed to more harmful side effects on themselves and their babies. The same reason was found for COVID-19 vaccine reluctance among pregnant/lactating mothers in previous studies [15,16]. One of the most common reasons for declining vaccination according to Sutton et al. included fear of harming the pregnancy [16].

Apart from the limitations related to online surveys, where only those who had access to the technology, resources, and ability to navigate the internet were approached and enrolled, the study included enrollment of women in any trimester of pregnancy and any time during the lactation period which limits the generalizability to each category. Although the number of respondents was not large, it was very diverse. Furthermore, vaccine acceptance levels among people might change over time as more data will be accrued.

## Conclusions

This study reported 56.2% acceptance of COVID-19 booster dose vaccination in a sample of pregnant/lactating women. Since booster vaccine against COVID-19 in pregnancy and during lactation can further control the burden of the disease and decrease morbidity and mortality during pregnancy/lactation, strategies to reduce vaccination hesitancy among the unvaccinated pregnant/lactating women as well as offering boosters to all already-vaccinated ones should be implemented to accomplish efficient COVID-19 immunization among this population. Relying on that and furthermore, vaccination trials in Jordan for pregnant/lactating women with a focus on short- and long-term safety and effectiveness data from vaccination during pregnancy and lactation periods are needed.

## Appendices

Mothers' impressions and beliefs about taking a booster dose for COVID-19 during pregnancy and lactation

Informed Consent Form

Dear participant

Researchers from different Jordanian Universities are carrying out a research project with the purpose to assess the acceptance of COVID-19 booster dose vaccination among pregnant and lactating women in Jordan.

Your participation in this research study is voluntary. You may choose not to participate. If you decide to participate in this research survey, you may withdraw at any time.

We would like to confirm that all information provided here will be kept confidential. Participant responses will be submitted directly through the website, and no personal identifiers will be collected.

The procedure involves filling an online survey that will take approximately 10 minutes. Your participation in completing this survey is highly appreciated.

Do you agree to participate in the study?

q Agree

q Disagree

Demographic data

Age: ____________________ (years)

Education level

q School

q Diploma

q Bachelor

q Master/PhD

Do you have a medical-related degree?

q Yes

q No

Personal income

q ≤500 JD/month 

q 501-1000 JD/month

q 1001-2000 JD/month 

q >2000 JD/month

Marital status

q Married

q Others

Nationality

q Jordanian

q Others

Place of residence

q South of Jordan 

q Middle of Jordan

q North of Jordan

Do you have any chronic comorbidity?

q Yes

q No

Pregnancy/Lactation status

q Pregnant

q Lactating

q Both

How many children do you have?

q 0

q 1

q 2

q 3

q 4+

Have you been infected and diagnosed with COVID-19 during pregnancy or lactation?

q Yes 

q No

Has someone pregnant/lactating you know been infected and diagnosed with COVID-19?

q Yes

q No

Up to this survey’s date, have you taken the vaccine before pregnancy?

q Yes, only the first dose

q Yes, both doses

q No

Up to this survey’s date, have you taken the vaccine while you are pregnant or lactating?

q Yes, only the first dose

q Yes, both doses

q No

 Did you have any side effects?

q No, I did not

q Yes, I had mild to moderate side effects

q Yes, I had severe side effects such as venous thromboembolism

q I did not receive the vaccine

General acceptance of vaccines

Do you commit with routine immunization for your children?

q Yes

q No

Do you think childhood vaccines are safe?

q Yes

q No

q Not sure

Do you think childhood vaccines are effective in protecting against diseases?

q Yes

q No

q Not sure

Did you receive any previous seasonal influenza vaccine?

q Yes

q No

q Not sure

Confidence in COVID-19 vaccine

When a COVID-19 vaccine is approved for pregnant and lactating women, how confident are you the booster dose will be safe and with no harmful effects?

q Not at all

q Not very

q Somewhat

q Fairly

q Very

When a COVID-19 vaccine is approved for pregnant and lactating women, how confident are you the booster dose will be effective and protect pregnant/lactating women who get it from getting COVID-19?

q Not at all

q Not very

q Somewhat

q Fairly

q Very

If COVID-19 vaccine were safe and available to pregnant/lactating women, how likely would you be to get a booster dose during pregnancy/lactating if the additional dose was effective?

q Not at all

q Quite unlikely

q Somewhat likely

q Fairly likely

q Very likely

Perceived risk of the virus/precautions

How worried are you about COVID-19 during pregnancy/lactation? (1 is low, 5 is high)

q 1

q 2

q 3

q 4

q 5

Do you think that you have perceived individual risk to catch COVID-19 during pregnancy/lactation?

q Yes

q No

q Not sure

Do you comply with your face mask and social distance guidelines?

q Always

q Usually

q Sometimes

q Rarely

q Never

Do you still monitor COVID-19 news on any media?

q Always

q Usually

q Sometimes

q Rarely

q Never

Acceptance to participate in a booster dose of COVID-19 vaccine clinical trial

Read the following definition the continue answering the below questions:

“COVID-19 clinical trial is a study that involved challenging participants with the virus after they develop immunity”

Are you willing to participate in COVID-19 booster dose vaccine clinical trial during pregnancy/lactation?

q Yes

q No

In your opinion, what are the motivation that may encourage you to participate in such clinical trials:

1. To help finding the best vaccine to COVID-19 during pregnancy/lactation

q Yes

q No

2. Vaccine might have specific side effects on pregnant/lactating women and should be tested in Jordan

q Yes

q No

3. I have relatives and friends who are pregnant/lactating that might suffer if vaccine was not developed

q Yes

q No

4. I might receive good compensation for my participation

q Yes

q No

In your opinion, what are the barriers that may discourage you to participate in such clinical trials:

1. I don’t like to volunteer in clinical studies either during pregnancy, lactation, or none

q Yes

q No

2. I don’t trust pharmaceutical companies that manufactured the vaccines

q Yes

q No

3. I don’t want to expose myself and the developing baby to any possible side effect

q Yes

q No

4. I have strong fear from the virus

q Yes

q No

5. My health status does not permit me to participate

q Yes

q No

6. I would like to see more safety and effectiveness data among pregnant/lactating women and their babies before participation

q Yes

q No

7. No need to conduct such studies in Jordan as the virus spread is under control

q Yes

q No

8. I have been/still am infected with coronavirus and thus cannot participate

q Yes

q No

9. I do not believe in vaccines in general

q Yes

q No
